# Population-attributable risk of psychiatric disorders for suicide among adolescents and young adults in Taiwan

**DOI:** 10.1017/S0033291722003361

**Published:** 2023-10

**Authors:** Yi-An Hung, Shih-Cheng Liao, Chia-Ming Chang, Shu-Sen Chang, Albert C. Yang, Yi-Ling Chien, Chi-Shin Wu, Susan Shur-Fen Gau

**Affiliations:** 1 Department of Psychiatry, National Taiwan University Hospital, Taipei, Taiwan; 2 Department of Psychiatry, College of Medicine, National Taiwan University, Taipei City, Taiwan; 3 Department of Psychiatry, National Taiwan University Hospital, Hsin-Chu Branch, Hsin-Chu Hospital, Hsin-Chu City, Taiwan; 4 Department of Psychiatry, Chang Gung Memorial Hospital at Linkou, Tao-Yuan, Taiwan; 5 Institute of Health Behaviors and Community Sciences, National Taiwan University, Taipei, Taiwan; 6 Digital Medicine Center / Institute of Brain Science, National Yang Ming Chiao Tung University, Taipei, Taiwan; 7 National Center for Geriatrics and Welfare Research, National Health Research Institutes, Zhunan, Taiwan; 8 Department of Psychiatry, National Taiwan University Hospital, Yunlin Branch, Yunlin, Taiwan; 9 Department of Psychology, Graduate Institute of Epidemiology, and Preventive Medicine, and Graduate Institute of Clinical Medicine, National Taiwan University, Taipei, Taiwan

**Keywords:** Adolescent, mental disorders, population-attributable risk, suicide, young adult

## Abstract

**Background:**

Youth suicide rates have increased markedly in some countries. This study aimed to estimate the population-attributable risk of psychiatric disorders associated with suicide among Taiwanese youth aged 10–24 years.

**Methods:**

Data were obtained from the National Death Registry and National Health Insurance (NHI) claims database between 2007 and 2019. Youth who died by suicide were included, and comparisons, 1:10 matched by age and sex, were randomly selected from the Registry for NHI beneficiaries. We used multivariable logistic regression to estimate suicide odds ratios for psychiatric disorders. The population-attributable fractions (PAF) were calculated for each psychiatric disorder.

**Results:**

A total of 2345 youth suicide and 23 450 comparisons were included. Overall, 44.8% of suicides had a psychiatric disorder, while only 7.9% of the comparisons had a psychiatric disorder. The combined PAF for all psychiatric disorders was 55.9%. The top three psychiatric conditions of the largest PAFs were major depressive disorder, dysthymia, and sleep disorder. In the analysis stratified by sex, the combined PAF was 45.5% for males and 69.2% for females. The PAF among young adults aged 20–24 years (57.0%) was higher than among adolescents aged 10–19 years (48.0%).

**Conclusions:**

Our findings of high PAF from major depressive disorder, dysthymia, and sleep disorder to youth suicides suggest that youth suicide prevention that focuses on detecting and treating mental illness may usefully target these disorders.

## Introduction

Suicide is a serious public health problem worldwide, with an estimated 700 000 deaths each year (World Health Organization, 2019). Despite numerous strategies for suicide prevention implemented over the years, suicide remains the third leading cause of death in 15- to 19-year-olds worldwide (World Health Organization, 2019). In Taiwan, suicide has long been one of the top five leading causes of death in the youth population, accounting for 7% of the total mortality in the 0–24 age group in recent years (Ministry of Health & Welfare, 2022). The suicide rate in the 0–24 age group has increased over the past ten years, from 2.6 per 100 000 in 2010 to 4.6 per 100 000 in 2019, with an increase of 77%. The lifetime prevalence of suicidal ideation, suicidal plans, and suicidal attempts among Taiwanese children were 8.2, 3.6, and 0.7%, respectively (Chen, Chen, Lin, Shen, & Gau, 2020). These studies highlight the importance of suicide prevention in young populations.

Investigating the factors associated with youth suicide is needed to develop specific strategies for youth suicide prevention. The identified risk factors include personality characteristics, psychiatric disorders, negative life experiences, physical health problems, parenting styles, and substance abuse (Gau et al., 2008). Among these, psychiatric disorders are recognised as one of the most important modifiable factors. Individuals with psychiatric disorders have a high relative risk of suicide ranging from 7 to 12 folds compared to those without psychiatric disorders (Li, Page, Martin, & Taylor, 2011).

However, most studies included all psychiatric disorders under the umbrella term of ‘psychopathology’ or ‘mental disorders’ or included only limited psychiatric diagnoses (Kline, Ortin-Peralta, Polanco-Roman, & Miranda, 2022; Page et al., 2014). Thus, the effects of distinct psychiatric disorders are unknown. In addition, the study samples of past studies were mostly individuals with suicidal ideation or suicidal attempts (Chan et al., 2009; Kline et al., 2022; Nock et al., 2013). The transition from suicidal ideation and suicidal attempts to actual suicide in youth is a highly complex process, and those who present with suicidal ideation and attempt and those who die by suicide might be very different vulnerable groups. Few studies have explicitly focused on the people who died by suicide (Benjet et al., 2018; Hill, Witt, Rajaram, McGorry, & Robinson, 2021; Lawrence et al., 2021; Page et al., 2014). Among risk factors of youth suicide, psychiatric disorders were known to have significant attribution to suicide and were potentially treatable and preventable. A more sophisticated focus on youth suicide may help understand the attribution of each specific psychiatric disorder and develop more targeted and effective strategies for prevention (Gould, Greenberg, Velting, & Shaffer, 2003).

In recent years, because suicide prevention has been prioritised in public health policies, more studies have estimated the impact of different modifiable risk factors on suicide (Gau et al., 2008; Hill et al., 2021; Page et al., 2014). The population-attributable fraction (PAF) is the estimated fraction of a particular disease in a population that would not have occurred in the absence of exposure. In this study, we used PAF to investigate the impact of each psychiatric disorder on suicide (Lin & Lu, 2008). In addition, adolescents and young adults are different in brain maturation, including emotional regulation, social cognition, and impulse control (Arain et al., 2013). Young adults also face social role transition, such as entering college or starting their first job, moving from their parent's house, and taking full law responsibility (Lenz, 2001). In addition, there are sex differences in the risk of suicide and psychiatric disorders (Miranda-Mendizabal et al., 2019). Therefore, we would explore the PAF of psychiatric disorders for suicide across adolescents and young adults as well as females and males.

## Methods

### Data source

We conducted a matched case–control study to estimate the risk of psychiatric disorders associated with suicide. Data were obtained from Taiwan's National Death Registry and the National Health Insurance Research Database between 2007 and 2019. Taiwan's National Death Registry includes detailed causes of death. All unnatural deaths, including suicide, homicide, or accidental death, were determined by a prosecutor (Ministry of Justice, 2021). The accuracy of suicide statistics was acceptable, based on one psychological autopsy study conducted in East Taiwan in the early 1990s that showed that 2 out of 117 suicides were misclassified as accidental deaths (Cheng, 1995). Suicides were identified using the International Classification of Disease, Ninth (ICD-9) and Tenth (ICD-10) Revisions, codes (ICD-9 code: E950–959; ICD-10 code: X60-X84 or X87.0).

The National Health Insurance Research Database (NHIRD) was derived from Taiwan's National Health Insurance (NHI) programme. Taiwan's government launched a universal and compulsory NHI programme in 1995, which included 99% of the Taiwanese population. The NHIRD, derived from the original claims data of the NHI programme, consists of the patient's demographic characteristics, clinical diagnoses, and prescription records. The accuracy of major psychiatric disorders is well documented (Wu, Kuo, Su, Wang, & Dai, 2020). The NHIRD can be linked to Taiwan's National Death Registry by the Data Science Centre of the Ministry of Health and Welfare of Taiwan; however, personal identification is anonymised for researchers to protect personal privacy.

### Study sample

Suicide cases aged between 10 and 24 years were identified based on the International Classification of Disease (ICD-9 code: E950–959 or ICD-10 code: X60-X84 or X87.0) in Taiwan's National Death Registry (Lin & Lu, 2008). For each suicide case, we randomly selected ten comparisons who did not die by suicide at the time of death and matched them with that case by age and sex from the Registry for NHI beneficiaries. The index date of the matched comparisons was defined by the date of suicide in the case group.

### Psychiatric disorders

We identified comorbid conditions of psychiatric disorders based on at least three ambulatory or one inpatient ICD diagnostic record before the index date of cases and comparisons (Wu, Lai, Gau, Wang, & Tsai, 2014). Psychiatric disorders included major depressive disorder, dysthymia, bipolar affective disorder, schizophrenia, substance or alcohol use disorder, any anxiety disorder, obsessive-compulsive disorder, sleep disorder, adjustment disorder, acute stress disorder/post-traumatic stress disorder, personality disorder, autism spectrum disorders, attention deficit hyperactive disorder (ADHD), tic disorders, oppositional defiant disorder/conduct disorder, and intellectual disability. Detailed ICD codes for psychiatric disorders are shown in online Supplementary Table S1.

### Statistical analysis

Descriptive statistics of suicide cases and comparisons are reported in terms of their demographic characteristics, including age, sex, monthly income, urbanicity (Liu et al., 2006), and calendar year of the index date. The prevalence of psychiatric disorders among suicide cases and comparisons were reported. To estimate each psychiatric disorder's adjusted odds ratio (aOR), we used multivariable logistic regression models with adjustment for age, sex, monthly income, urbanicity, calendar year of the index date, and all the above-mentioned psychiatric disorders simultaneously.

The proportion of suicide that could be attributed to a particular psychiatric disorder, compared with the absence of this disorder, was determined using the PAF, which was calculated using the following equation:
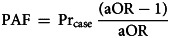
where Pr_case_ is the prevalence of suicide cases diagnosed with a specific psychiatric disorder, and aOR is the previously calculated aOR of suicide. The combined PAF of all psychiatric disorders for suicide was calculated using the following formula:
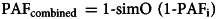
(Rockhill, Newman, & Weinberg, 1998)

Subgroup analyses were conducted to explore the PAFs of psychiatric disorders by sex and age group, including adolescents (aged 10–19 years) and young adults (aged 20–24 years). The above analysis was performed using SAS version 9.4; a 95% confidence interval (CI) and a *p* value < 0.05 indicated statistical significance for all analyses.

## Results

Table 1 presents the demographic characteristics of the 2345 cases of suicide and 23 450 comparisons, matched by year of birth, sex, and calendar year. In total, 64.1% of the subjects were male. Most subjects were between 20 and 24 years of age (73.9%). Approximately half of the subjects lived in urban areas, while less than 10% lived in rural areas. The distribution of urbanicity in both groups was not significantly different (*p* = 0.197). However, subjects who died by suicide had a lower monthly income than the comparisons (*p* < 0.001).

**Table 1.** Baseline characteristics of patients who died by suicide and comparisons

	Suicide group (*n* = 2345)	Comparison group (*n* = 23 450)	*p* value
Age			NA
10-14	62 (2.6)	620 (2.6)	
15-19	549 (23.4)	5490 (23.4)	
20-24	1734 (73.9)	17 340 (73.9)	
Sex			NA
Male	1504 (64.1)	15 040 (64.1)	
Female	841 (35.9)	8410 (35.9)	
Urbanicity			0.197
Urban	1240 (52.9)	11 960 (51.0)	
Suburban	889 (37.9)	9171 (39.1)	
Rural	216 (9.2)	2319 (9.9)	
Monthly income			<0.001
< 19 200	998 (42.6)	5998 (25.6)	
19 200– 34 999	986 (42.0)	13 198 (56.3)	
⩾35 000	361 (15.4)	4254 (18.1)	
Calendar year			NA
2007	194 (8.3)	1940 (8.3)	
2008	193 (8.2)	1930 (8.2)	
2009	178 (7.6)	1780 (7.6)	
2010	155 (6.6)	1550 (6.6)	
2011	160 (6.8)	1600 (6.8)	
2012	181 (7.7)	1810 (7.7)	
2013	152 (6.5)	1520 (6.5)	
2014	144 (6.1)	1440 (6.1)	
2015	165 (7.0)	1650 (7.0)	
2016	191 (8.1)	1910 (8.1)	
2017	182 (7.8)	1820 (7.8)	
2018	200 (8.5)	2000 (8.5)	
2019	250 (10.7)	2500 (10.7)	

Overall, 44.8% of subjects who died by suicide had at least a psychiatric disorder, while only 7.9% of the comparisons had been diagnosed with any psychiatric disorder. Table 2 shows the prevalence, crude and aORs, and PAF of each psychiatric disorder for suicide. Among cases of suicide, psychiatric disorders with a prevalence of > 10% were major depressive disorder (20.3%), anxiety disorder (19.7%), sleep disorder (18.0%), and dysthymia (16.8%). Among the comparison group, the prevalence of psychiatric disorders was below 4%, with three psychiatric disorders having the highest prevalence: any anxiety disorder (3.1%), sleep disorder (2.4%), and ADHD (1.3%). Schizophrenia (aOR 8.60; 95% CI 6.03–12.27), Substance or alcohol use disorder (aOR 8.14; 95% CI 5.44–12.16), and major depressive disorder (aOR 6.09; 95% CI 4.90–7.57) were highly associated with suicide. Although the crude odds ratios were significantly greater than one, the associations between suicide and intellectual disability (aOR 0.61; 95% CI 0.38–0.97) were inverse.

**Table 2.** Prevalence, odds ratios, and population attributable fractions of psychiatric disorders for suicide among adolescents and young adults

	Case with suicide (*n* = 2345)	Comparisons (*n* = 23 450)	Crude ORs (95% CI)	aORs (95% CI)	Population Attributable Fraction, %
Psychiatric disorder					
Major depressive disorder	475 (20.3)	218 (0.9)	27.07 (22.90–31.99)	6.09 (4.90–7.57)	16.93
Dysthymia	393 (16.8)	244 (1.0)	19.15 (16.21–22.61)	3.64 (2.89–4.57)	12.15
Bipolar affective disorder	188 (8.0)	78 (0.3)	26.12 (19.98–34.13)	2.32 (1.60–3.38)	4.57
Schizophrenia	177 (7.5)	63 (0.3)	30.31 (22.66–40.54)	8.60 (6.03–12.27)	6.67
Substance or alcohol use disorder	150 (6.4)	51 (0.2)	31.35 (22.75–43.21)	8.14 (5.44–12.16)	5.61
Any anxiety disorder	463 (19.7)	737 (3.1)	7.58 (6.69–8.59)	1.87 (1.55–2.26)	9.20
Obsessive-compulsive disorder	61 (2.6)	45 (0.2)	13.89 (9.43–20.47)	1.38 (0.79–2.41)	0.71
Sleep disorder	421 (18.0)	562 (2.4)	8.91 (7.79–10.20)	2.19 (1.80–2.67)	9.76
Adjustment disorder	204 (8.7)	205 (0.9)	10.80 (8.86–13.18)	2.17 (1.64–2.87)	4.69
Acute stress disorder/Post-traumatic stress disorder	57 (2.4)	62 (0.3)	9.40 (6.54–13.50)	1.51 (0.87–2.60)	0.82
Personality disorder	128 (5.5)	36 (0.2)	37.55 (25.88–54.49)	2.58 (1.56–4.27)	3.35
Attention deficit hyperactivity disorder	113 (4.8)	314 (1.3)	3.73 (3.00–4.65)	1.49 (1.09–2.04)	1.58
Tic disorders	17 (0.7)	58 (0.2)	2.95 (1.71–5.07)	1.00 (0.47–2.09)	0.00
Oppositional defiant disorder/Conduct disorder	33 (1.4)	43 (0.2)	7.77 (4.93–12.25)	1.99 (1.05–3.77)	0.70
Autism spectrum disorders	39 (1.7)	60 (0.3)	6.59 (4.40–9.89)	1.81 (0.99–3.33)	0.75
Intellectual disability	48 (2.0)	189 (0.8)	2.57 (1.87–3.54)	0.61 (0.38–0.97)	−1.34

aORs, adjusted odds ratios.

Overall, the combined PAF rate for all psychiatric disorders was 55.9%. The highest proportion of suicides was attributable to major depressive disorder (PAF = 16.93%), followed by dysthymia (PAF = 12.15%), sleep disorders (PAF = 9.76%), anxiety disorders (PAF = 9.20%), schizophrenia (PAF = 6.67%), and Substance or alcohol use disorder (PAF = 5.61%). The remaining psychiatric disorders had low PAF values (<5%).

Table 3 shows the prevalence, aOR, and PAFs of each psychiatric disorder for suicide by sex. Overall, the combined PAF was 45.5% in men and 69.2% in women. Psychiatric disorders with the three highest aOR were the same in both sexes, including major depressive disorder, schizophrenia, and Substance or alcohol use disorder. However, the degree to which they were associated with suicide differed significantly. The aOR for Substance or alcohol use disorder was higher in men, while the aOR for schizophrenia was higher in women. Major depressive disorder had the highest PAF in both sexes, but the attribution was much higher in female youth suicide. In addition, the PAF of dysthymia, sleep disorder, and any anxiety disorder were higher than those of other psychiatric disorders in both sexes; however, the point estimate of PAFs was generally higher for women than that for men.

**Table 3.** Prevalence, adjusted odds ratios, and population attributable fractions of psychiatric disorders for suicide among adolescents and young adults, by sex

	Male				Female			
	Case with suicide (*n* = 1504)	Comparisons (*n* = 15 040)	aORs (95% CI)	Population Attributable Fraction, %	Case with suicide (*n* = 841)	Comparisons (*n* = 8410)	aORs (95% CI)	Population Attributable Fraction, %
Psychiatric disorder								
Major depressive disorder	234 (15.6)	121 (0.8)	5.66 (4.24–7.55)	12.81	241 (28.7)	97 (1.2)	7.03 (5.01–9.86)	24.58
Dysthymia	180 (12.0)	132 (0.9)	2.87 (2.09–3.94)	7.79	213 (25.3)	112 (1.3)	4.67 (3.33–6.55)	19.91
Bipolar affective disorder	97 (6.4)	52 (0.3)	2.11 (1.31–3.41)	3.40	91 (10.8)	26 (0.3)	2.69 (1.45–5.00)	6.80
Schizophrenia	101 (6.7)	43 (0.3)	7.06 (4.52–11.03)	5.76	76 (9.0)	20 (0.2)	13.97 (7.68–25.42)	8.39
Substance or alcohol use disorder	90 (6.0)	32 (0.2)	10.77 (6.59–17.59)	5.43	60 (7.1)	19 (0.2)	4.78 (2.37–9.61)	5.64
Any anxiety disorder	234 (15.6)	400 (2.7)	1.86 (1.45–2.39)	7.20	229 (27.2)	337 (4.0)	1.82 (1.36–2.45)	12.30
Obsessive–compulsive disorder	37 (2.5)	36 (0.2)	1.24 (0.64–2.39)	0.47	24 (2.9)	9 (0.1)	2.40 (0.78–7.42)	1.66
Sleep disorder	211 (14.0)	298 (2.0)	2.26 (1.74–2.94)	7.82	210 (25.0)	264 (3.1)	2.17 (1.60–2.94)	13.44
Adjustment disorder	106 (7.0)	134 (0.9)	1.70 (1.18–2.45)	2.90	98 (11.7)	71 (0.8)	3.38 (2.16–5.31)	8.21
Acute stress disorder/Post-traumatic stress disorder	30 (2.0)	31 (0.2)	1.72 (0.83–3.55)	0.83	27 (3.2)	31 (0.4)	1.32 (0.57–3.03)	0.77
Personality disorder	51 (3.4)	24 (0.2)	2.12 (1.06–4.25)	1.79	77 (9.2)	12 (0.1)	3.23 (1.46–7.12)	6.32
Attention deficit hyperactivity disorder	91 (6.1)	267 (1.8)	1.57 (1.11–2.22)	2.19	22 (2.6)	47 (0.6)	1.31 (0.60–2.84)	0.61
Tic disorders	13 (0.9)	53 (0.4)	1.00 (0.46–2.17)	0.00	4 (0.5)	5 (0.1)	1.46 (0.15–14.42)	0.15
Oppositional defiant disorder/Conduct disorder	27 (1.8)	35 (0.2)	2.68 (1.37–5.23)	1.12	6 (0.7)	8 (0.1)	0.33 (0.05–2.01)	−1.45
Autism spectrum disorders	32 (2.1)	54 (0.4)	1.91 (1.02–3.59)	1.01	7 (0.8)	6 (0.1)	2.52 (0.38–16.75)	0.50
Intellectual disability	32 (2.1)	155 (1.0)	0.50 (0.29–0.87)	−2.09	16 (1.9)	34 (0.4)	1.55 (0.59–4.09)	0.68

aORs, adjusted odds ratios.

Table 4 shows the PAFs of each psychiatric disorder for suicide among adolescents and young adults. Overall, the combined PAF for all psychiatric disorders was 48.0% in adolescents and 57.0% in young adults. Psychiatric disorders with the highest aOR in both groups were schizophrenia, Substance or alcohol use disorder, and major depressive disorder. However, compared with young adults, the magnitude of the association of these three disorders with suicide was significantly higher in adolescents. Another notable difference was that adolescents with obsessive-compulsive disorder had a much higher aOR and PAF. However, among young adults, obsessive-compulsive disorder was not significantly associated with suicide. In both age groups, major depressive disorder, dysthymia, and any anxiety disorder had relatively similar PAF values. Notably, sleep disorders contributed significantly to suicide in young adults, which was not observed in adolescents. In young adults, intellectual disability was negatively associated with suicide.

**Table 4.** Prevalence, odds ratios, and population attributable fractions of psychiatric disorders for suicide among adolescents and young adults, by age

	Age 10–19 years				Age 20–24 years			
	Case with suicide (*n* = 1504)	Comparisons (*n* = 15 040)	aORs (95% CI)	Population Attributable Fraction, %	Case with suicide (*n* = 841)	Comparisons (*n* = 8410)	aORs (95% CI)	Population Attributable Fraction, %
Psychiatric disorder								
Major depressive disorder	100 (16.4)	21 (0.3)	16.66 (9.36–29.64)	15.38	375 (16.0)	197 (0.8)	5.26 (4.13–6.69)	17.51
Dysthymia	80 (13.1)	29 (0.5)	6.93 (3.89–12.32)	11.20	313 (13.3)	215 (0.9)	3.27 (2.54–4.22)	12.53
Bipolar affective disorder	31 (5.1)	7 (0.1)	2.13 (0.59–7.72)	2.69	157 (6.7)	71 (0.3)	2.45 (1.65–3.63)	5.35
Schizophrenia	34 (5.6)	3 (0.0)	30.49 (8.11–114.56)	5.38	143 (6.1)	60 (0.3)	8.07 (5.54–11.77)	7.23
Substance or alcohol use disorder	18 (2.9)	6 (0.1)	16.43 (5.50–49.08)	2.77	132 (5.6)	45 (0.2)	7.65 (4.97–11.78)	6.62
Any anxiety disorder	86 (14.1)	102 (1.7)	2.07 (1.31–3.29)	7.29	377 (16.1)	635 (2.7)	1.83 (1.49–2.25)	9.86
Obsessive-compulsive disorder	19 (3.1)	6 (0.1)	7.81 (2.28–26.78)	2.71	42 (1.8)	39 (0.2)	0.96 (0.51–1.81)	−0.10
Sleep disorder	57 (9.3)	62 (1.0)	1.23 (0.67–2.25)	1.74	364 (15.5)	500 (2.1)	2.41 (1.95–2.97)	12.28
Adjustment disorder	52 (8.5)	45 (0.7)	2.25 (1.22–4.13)	4.72	152 (6.5)	160 (0.7)	2.12 (1.54–2.94)	4.64
Acute stress disorder/Post-traumatic stress disorder	9 (1.5)	12 (0.2)	1.11 (0.30–4.17)	0.14	48 (2.0)	50 (0.2)	1.56 (0.85–2.87)	1.00
Personality disorder	16 (2.6)	3 (0.0)	2.58 (0.45–14.77)	1.60	112 (4.8)	33 (0.1)	2.88 (1.70–4.90)	4.22
Attention deficit hyperactivity disorder	60 (9.8)	170 (2.8)	1.58 (0.99–2.52)	3.60	53 (2.3)	144 (0.6)	1.12 (0.70–1.78)	0.32
Tic disorders	11 (1.8)	31 (0.5)	1.66 (0.69–4.00)	0.72	6 (0.3)	27 (0.1)	0.30 (0.08–1.16)	−0.79
Oppositional defiant disorder/Conduct disorder	16 (2.6)	22 (0.4)	1.81 (0.73–4.53)	1.18	17 (0.7)	21 (0.1)	1.64 (0.62–4.31)	0.38
Autism spectrum disorders	16 (2.6)	21 (0.3)	1.58 (0.56–4.45)	0.96	23 (1.0)	39 (0.2)	1.88 (0.85–4.16)	0.62
Intellectual disability	17 (2.8)	54 (0.9)	1.24 (0.55–2.76)	0.53	31 (1.3)	135 (0.6)	0.47 (0.26–0.83)	−2.05

aORs, adjusted odds ratios.

## Discussion

### Main findings

In this study, we found that 44.8% of adolescents and young adults who died by suicide had at least one psychiatric diagnosis, whereas only 7.9% of the comparisons had psychiatric diagnoses. Overall, psychiatric disorders were attributable to 55.2% of the youth suicides. Major depressive disorder, dysthymia, and sleep disorder are the three major psychiatric disorders that contribute to youth suicides. In terms of subgroup analysis, we found that the PAF of overall psychiatric disorders for suicide among women (69.2%) was higher than that among men (45.5%). PAF among young adults (57.0%) was higher than that among adolescents (48.0%).

Our findings are in line with one cross-sectional study using coronial information in Australia, which reported that 40.9% of young people who died by suicide had a diagnosed mental disorder, and 15.7% had possible mental disorders (Hill et al., 2021). A psychological autopsy study conducted in the United States showed that 59% of 120 subjects aged < 20 years who died by suicide had psychiatric diagnoses (Shaffer et al., 1996). Another study using the Demark Cause-of-Death Register and Psychiatric Center Register found that 38.7% of men and 57.1% of women aged ⩽ 35 years who died by suicide had a history of psychiatric admission (Qin, 2011). Another case–control Australian study using coronial information for 84 youth suicides aged 18–34 years showed that the combined PAF for all psychiatric disorders was 48% in men and 52% in women (Page et al., 2014). All these studies demonstrate that the prevalence of psychiatric disorders among suicide cases is much higher than that of comparisons, suggesting that psychiatric disorders account for a substantial proportion of suicides.

The increasing trend in youth suicide is seen in Taiwan and many parts of the world, including the United Kingdom, the United States, Canada, and Australia (Chang et al., 2022b; Padmanathan, Bould, Winstone, Moran, & Gunnell, 2020). In Taiwan, elevated incident depressive disorders among adolescents and young adults (Wang et al., 2022) might explain the increasing trend of youth suicide. Although the mental healthcare resource increased during the study periods (Wang et al., 2022), the treatment rate was still low. We found that the prevalence of treated psychiatric disorders from this claims-based study was much lower than that of a nationwide survey, which showed the lifetime prevalence of psychiatric disorders among children and early adolescents was 31.6% (Chen et al., 2020). In addition, income inequality, elevated parental divorce rates, increased prevalence of Internet use and poor sleep were also associated with the increasing suicide trends (Chang et al., 2022b; Padmanathan et al., 2020). The links between these social factors and suicide might be partially mediated by developing psychiatric disorders.

### Depressive disorders and suicide

Depressive disorders, including major depressive disorder and dysthymia, contributed to the highest proportion of youth suicide in this study, mainly due to the high prevalence across sex and age groups. The underlying mechanism of the association between depression and suicide is complex, including the synergic effects of psychosocial stressors, genetics, monoaminergic neurotransmitters, and other neuromodulators (Orsolini et al., 2020). Many symptoms included in the diagnostic criteria of depressive disorders are associated with increased suicidal risks, including concentration deficit, poor sleep, low self-esteem, worthlessness, and negative inferential style (Burke et al., 2016; Nrugham, Larsson, & Sund, 2008). Anhedonia, also known as the inability to experience pleasure, is greater among adolescent suicide attempters than suicidal ideators or control (Auerbach, Millner, Stewart, & Esposito, 2015), and is predictive of subsequent suicidal events (Yen et al., 2013). Although the male gender is a risk factor for suicide among patients with depression; a recent meta-analysis study demonstrated that the suicidal risk of affective disorder was similar in both sexes (Li et al., 2011). In our study, the aORs of depressive disorders for suicide were similar between women and men. Given that the prevalence of depressive disorder was much higher among women than among men, the PAF of depressive disorder in women was 2-fold higher than that in men. Furthermore, we found the odds ratio of depressive disorder among adolescents was much higher than that among young adults. One Denmark study also showed that the incident rate ratios of depression for suicide were inversely associated with age (Qin, 2011). These findings might partially explain the increased suicide risk of antidepressant treatment among the young population, especially adolescents (Esang & Ahmed, 2018).

### Schizophrenia and suicide

Patients with schizophrenia had the highest aOR among the overall study sample. In terms of subgroup analysis, we found that suicide risk among women was higher than that among men. One Demark study (Qin, 2011) and one systematic review (Li et al., 2011) also demonstrated sex differences in the suicide risk associated with schizophrenia, with an increased risk for suicide in schizophrenia 10 times for males and 20 times for females. A possible explanation is that the symptom profile is different across sexes; men score higher in negative symptoms and disorganisation, and women have a higher prevalence of depressive symptoms (Morgan, Castle, & Jablensky, 2008). Depressive symptoms were the most important factor related to suicidality (Barbeito et al., 2021). We also found that the suicide risk was higher among adolescents than young adults. A possible explanation is that suicide often occurs in the early course of schizophrenia, resulting in high suicide risk among adolescents (Moe et al., 2022).

### Substance or alcohol use disorder and suicide

Substance or alcohol use disorder had the second-highest aOR for youth suicide. The association between suicide and substance or alcohol use disorder has been widely demonstrated (Esang & Ahmed, 2018). Possible explanations include that substance or alcohol use would weaken impulse control and impair judgment, thereby increasing the risk of committing suicide (Albert, De Ronchi, Maina, & Pompili, 2019). There was no overt sex difference in the association between substance or alcohol use disorders and suicide. We found that the risk of substance or alcohol use disorder for suicide is high among adolescents, which indicated that adolescents were vulnerable to substance and alcohol (Pompili et al., 2012). In addition, the legal alcohol drinking age ranges from 18 to 21 in most countries; thus, the prevalence of alcohol use disorder increased dramatically among young adults. Overall, the PAF of substance or alcohol use disorder among adolescents and young adults was similar.

### Anxiety disorder and suicide

The suicide risk of anxiety disorder was relatively low compared to other psychiatric disorders, while its PAF was near 10%, owing to its high prevalence. The prevalence of anxiety disorder in women was almost twice that in men, which corresponds with a large Denmark population study of 21 169 suicides (Qin, 2011). Previous studies have shown that anxiety disorder is associated with a higher risk of youth suicide (Page et al., 2014; Qin, 2011), even after controlling for comorbid affective disorders and demographic characteristics. Possible explanations include difficulty maintaining interpersonal relationships when interpreting and coping with emotions.

### Sleep disorder and suicide

Sleep disorders are prevalent in youth. It was the second most common psychiatric disorder in the comparison group and the third in the suicide group. Several studies have demonstrated that sleep disorders are associated with high suicidality among youth compared with that of other age groups (Liu et al., 2019; Wong, Brower, & Craun, 2016). Although sleep disorders might often be comorbid with other psychiatric disorders, it was a significant independent risk factor after controlling for covariates such as substance or alcohol use disorder or mood disorders (Goldstein, Bridge, & Brent, 2008; Liu et al., 2019; Wong et al., 2016). One possible explanation is that sleep disturbance worsens problem-solving abilities and increases impulsivity due to sleep deprivation and disrupted frontal lobe function (Goldstein et al., 2008; Perlis et al., 2016). Another explanation is that sleep disturbance leads to low serotonin synthesis and postsynaptic serotonin receptor sensitisation in the prefrontal cortex (Liu et al., 2019), finally resulting in poor judgment, increased impulsivity, and aggression.

### Obsessive-compulsive disorder and suicide

Recent research had shown that OCD was significantly associated with suicidality, even after adjusting for socio-demographic variables and comorbidities (Albert et al., 2019; Angelakis, Gooding, Tarrier, & Panagioti, 2015). However, there remained wide variation in prevalence and odds ratios between different studies (Albert et al., 2019; Angelakis et al., 2015; Pellegrini et al., 2020). It might be due to limitations, including high heterogeneity of study groups, high comorbidities with other psychiatric disorders, and different instruments used to assess suicidality (Pellegrini et al., 2020). In addition, most studies were on suicidal ideation and attempts, except for two population-based studies from Sweden and Denmark, demonstrating that patients with OCD had an increased risk of suicide and unnatural death, respectively (Fernández de la Cruz et al., 2017; Meier et al., 2016). In our study, only adolescents with OCD had a significantly increased risk of suicide. The association between OCD and suicide was not statistically significant among young adults. Whether there is an effect modification of age on the link between OCD and suicide needs future investigation.

### Intellectual disability and suicide

The finding of reduced suicide risk in intellectual disability was consistent with studies in different nations. One retrospective cohort study from Denmark reported lower suicidal behaviours in intellectual disability (Erlangsen et al., 2020). Another Finnish study demonstrated that men with intellectual disabilities have a lower suicide risk (Patja, Iivanainen, Raitasuo, & Lönnqvist, 2001). People with intellectual disabilities might have limited skills in assessing high fatality suicide methods, thereby reducing suicide risk (Patja et al., 2001). The severity of intellectual disability might also have a different influence on suicidality. Further research is needed to investigate the related mechanism or factors involved.

### Psychiatric disorders with minor impact on suicide

Most psychiatric disorders were associated with a higher risk of youth suicide, except for tic disorders, autism, and acute stress disorder/post-traumatic stress disorder. The association between tic disorder and suicide was insignificant after controlling for other comorbid psychiatric disorders, indicating that tic disorders might not be an independent risk factor. The case number of acute stress disorder/post-traumatic stress disorder was relatively small, which might be because under-recognised in youth is common. Furthermore, ADHD and oppositional defiant disorder/conduct disorder were associated with an elevated risk of both OR and PAF in adolescents. The severity of these disorders generally subsided in adulthood; therefore, their association with suicide was not found in young adults. Notably, the prevalence of ADHD in our suicidal and non-suicidal groups was still much lower than those found in past Taiwanese epidemiological studies (Chen et al., 2020). This result could be explained by the fact that ADHD is still underdiagnosed and undertreated in Taiwan. The impact of ADHD on suicide risk may have been underestimated in the present study.

### Strengths and limitations

The strengths of this study are the use of the National Death Registry to identify all youth suicide and the comprehensive assessment of psychiatric disorders from the NHI outpatient and inpatient claims records. In addition, subgroup analyses were conducted to explore the modifying effects of age and sex. However, our study has several limitations. First, we assessed psychiatric diagnoses using the claims records. Individuals with psychiatric problems who did not seek help were not included. Previous epidemiological studies in Taiwan suggested that approximately 20–30% of individuals with depression sought medical treatment (Chang et al., 2022a; Liao et al., 2012). Therefore, the prevalence of psychiatric disorders has been underestimated. Generally, women are more likely to seek treatment than men; therefore, this might partly explain the sex difference in PAF of psychiatric disorders for suicide (Chang, 2007; Miranda-Mendizabal et al., 2019). In addition, individuals with lower incomes have more barriers to assessing mental healthcare services (Magaard, Seeralan, Schulz, & Brütt, 2017). Low income and other socioeconomic disadvantages were associated with poor mental health, such as a high risk of developing psychiatric disorders and high suicidality (Islam, Ormsby, Kabir, & Khanam, 2021). Suicide group had significantly lower income than the comparison group; therefore, the odds ratios and PAF for suicide might be underestimated. Second, our database did not include several important confounding factors, such as childhood adversity, family support, family history of psychiatric disorders and suicide, and psychosocial stressors. Thus, the magnitude of the association between psychiatric disorders and suicide may be overestimated by not accounting for these factors. Third, we did not explore the odds ratio and PAF of specific psychiatric comorbidity for youth suicide because the possible combinations of psychiatric disorders are numerous, and the study sample size was limited. Therefore, we estimated the odds ratios and PAF of each psychiatric disorder for suicide with adjustment for other psychiatric disorders. The methods were consistent with previous studies (Chan et al., 2009; Page et al., 2014). Fourth, we did not further distinguish early and late adolescents in subgroup analysis because the suicide number in early adolescence, aged between 10–14 years, was small (*n* = 62). Further investigations are needed to explore the role of psychiatric disorder on suicide among early adolescent. Finally, we calculated the PAFs of psychiatric disorders for suicide under the assumption that all psychiatric disorders could be treated completely. However, not all psychiatric disorders can be completely prevented or treated. Thus, the number of preventable cases may be lower than our estimates using PAFs.

### Clinical implication

Our findings emphasise that early recognising and treating psychiatric disorders for youth suicide prevention, as more than half of youth suicide could be attributed to psychiatric disorders. School-based staff education and screening might help detect youth's psychiatric disorders and suicide risks early (Anderson et al., 2019; Singer, Erbacher, & Rosen, 2019; Zalsman et al., 2016). Evidence shows that psychotherapy and psychosocial intervention could treat psychiatric disorders and reduce suicide risk (Calear et al., 2016; Zalsman et al., 2016). Although antidepressant agents effectively reduce depressive and anxiety symptoms, they were associated with increasing suicidality (Friedman & Leon, 2007) and should be used cautiously in the youth.

Public health resources are often limited, so focusing on disorders with higher PAF, including major depressive disorder, dysthymia, sleep disorder, and anxiety disorders, might be more efficient. Contrary to the common belief that men with schizophrenia had high suicidality, our study revealed that women with schizophrenia had higher suicide risks and required more assessment and management. Interestingly, sleep disorders are shown to have a significant role in youth suicide. This sheds light on the importance of evaluating sleep quality and quantity in youth. In primary care settings, youth with sleep disturbance should be assessed carefully, especially for the potential comorbid psychiatric conditions and suicide risk.

Although psychiatric disorders were the most important risk factor, other factors were still attributable to approximately half of the youth suicides. Restricting access to lethal means, establishing media guidelines for reporting suicide, gatekeeper training, follow-up contact for suicidal attempters, and other strategies should be combined to prevent youth suicide (Zalsman et al., 2016).

### Future research implication

As our data is generated from the NHI claims database, several important confounding factors are unavailable. Future studies should consider these factors, including childhood adversity, family support, and psychosocial stressors. In addition, a family history of suicide or psychiatric disorder could play an important role in youth suicide (Agerbo, Nordentoft, & Mortensen, 2002; Campos, Holden, Spínola, Marques, & Santos, 2020). Further investigations should address the impact of family history on the risk of youth suicide. Moreover, the treatment rate of psychiatric disorders in youth is still low. A proportion of non-diagnosed subjects who died by suicide may likely include those with undiscovered mental illness. Barriers to mental health service utilisation in youth should also be explored. Finally, the effectiveness of youth suicide prevention strategies is needed to be evaluated for the extent to which treatment can decrease youth suicide.

## Conclusion

The youth suicide rate has been increasing in many countries, including Taiwan. Psychiatric disorders have a high prevalence in youth and are strongly associated with youth suicide. Major depressive disorders, dysthymia, and sleep disorders were shown to have high PAF and, therefore, should be the main targets for future youth suicide prevention. Enhancing early identification and effective treatment should be the primary strategies for suicide prevention in youth.

## Supplementary material

For supplementary material accompanying this paper visit https://doi.org/10.1017/S0033291722003361. click here to view supplementary material
