# Supplementary material for: Population-attributable risk of psychiatric disorders for suicide among adolescents and young adults in Taiwan
Source: Psychol Med. 2022 Nov 9;53(13):6161–70. doi: 10.1017/S0033291722003361 (PMC10520582; doi:10.1017/S0033291722003361)
Supplement: Supplementary file 1 [file S0033291722003361sup001.docx]

Supplementary Table 1. ICD codes used for identifying psychiatric diagnoses

| **Psychiatric Disorder** | **ICD-9** | **ICD-10** |
| --- | --- | --- |
| Major depressive disorder | 296.2; 296.3 | F32, F33 |
| Dysthymia | 300.4 | F34.1 |
| Bipolar affective disorder | 296.0, 296.1, 296.4, 296.5, 296.6, 296.7, 296.8 | F30, F31 |
| Schizophrenia | 295 | F20; F25 |
| Substance or alcohol use disorder | 291, 292, 303, 304, 305.0, 305.2-305.9 | F10-F16, F18, F19 |
| Any anxiety disorder | 300.0, 300.2 | F40, F41 |
| Obsessive-compulsive disorder | 300.3 | F42 |
| Sleep disorder | 307.4, 780.5 | G47, F51 |
| Adjustment disorder | 309.0-309.4 | F43.2 |
| Acute stress disorder/Post-traumatic stress disorder | 308, 309.81 | F43.0, F43.1 |
| Personality disorder | 301 | F60 |
| Attention deficit hyperactivity disorder | 314 | F90 |
| Tic disorders | 307.2 | F95 |
| Oppositional defiant disorder/Conduct disorder | 312, 313.81 | F91, F93 |
| Autism spectrum disorders | 299 | F84 |
| Intellectual disability | 317-319 | F70-F73, F78, F79 |
